# Supplementary material for: Community use of oral antibiotics transiently reprofiles the intestinal microbiome in young Bangladeshi children
Source: Nat Commun. 2024 Aug 14;15:6980. doi: 10.1038/s41467-024-51326-5 (PMC11324872; doi:10.1038/s41467-024-51326-5)
Supplement: Supplementary file 1 — Supplementary Information [file 41467_2024_51326_MOESM1_ESM.pdf]

Supplementary Information

Figure S1

Violin plots illustrating taxonomic alpha diversity between 8 and 20 months of age (16S rRNA data). n = 900 at 8 months, 792 at 11 months and 574 at 20 months.

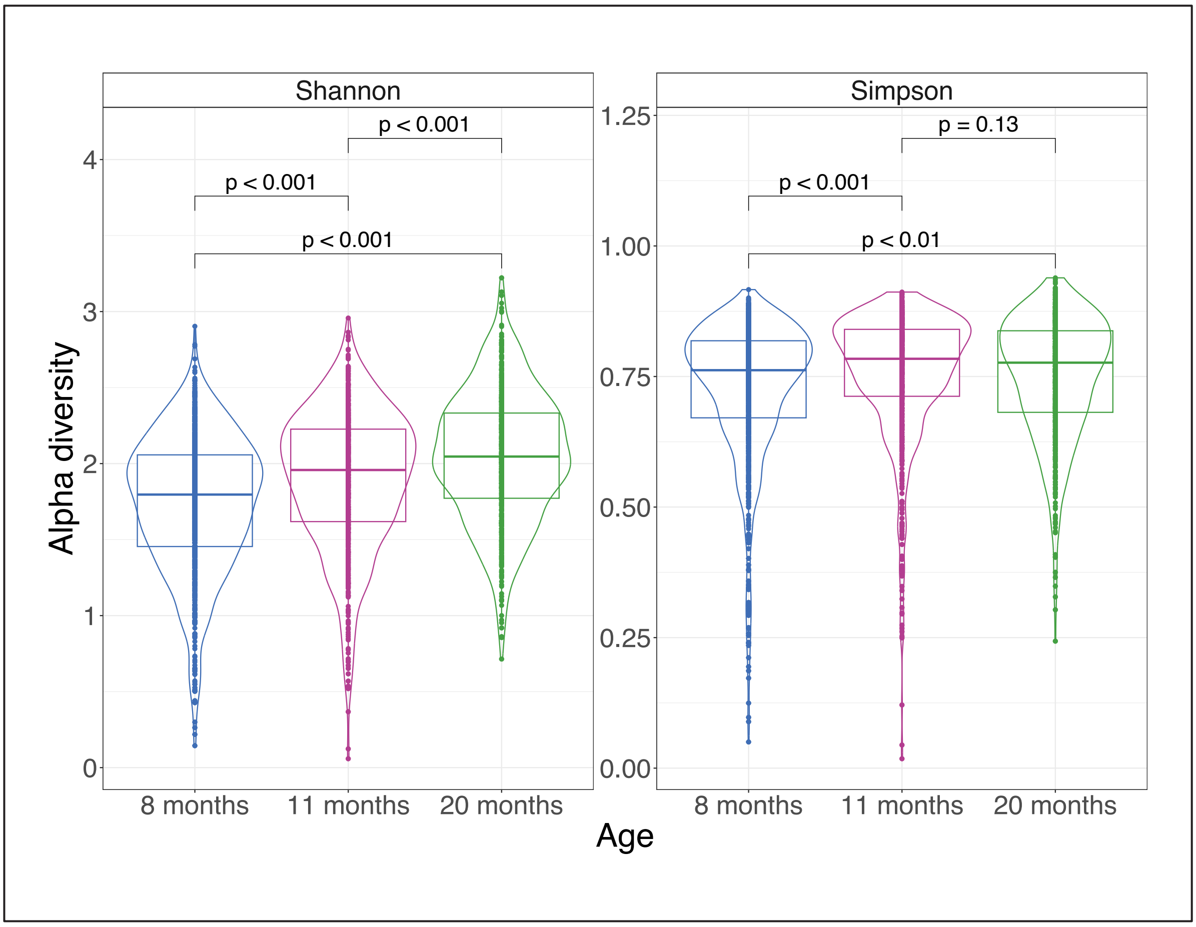

Shannon
Simpson
p < 0.001
p < 0.001
p < 0.001
p = 0.13
p < 0.001
p < 0.01
Alpha diversity
0
1
2
3
4
0.00
0.25
0.50
0.75
1.00
1.25
8 months
11 months
20 months
8 months
11 months
20 months
Age

Figure S2

A: Violin plots illustrating taxonomic alpha diversity in samples at the midline timepoint by antibiotic use anytime during the intervention (401 with antibiotic use from a total of 792) (16S rRNA data). **B-C**. Volcano plots illustrating differential abundance of species (**B**) and AMR genes (**C**) in relation to antibiotic use anytime during the intervention (180 with antibiotic use out of 316 for both analyses) (Shotgun metagenomic data). **B** and **C** show $\log_2$-fold change on the x-axis and the $-\log_{10}$(FDR-adjusted *P*-value) on the y-axis. The horizontal red line marks the level equivalent to an adjusted *P*-value 0.05. AMR gene abundance was measured in reads per kilobase per million sample reads (RPKM). Source data for Figure S2B-C are provided in the Source Data file.

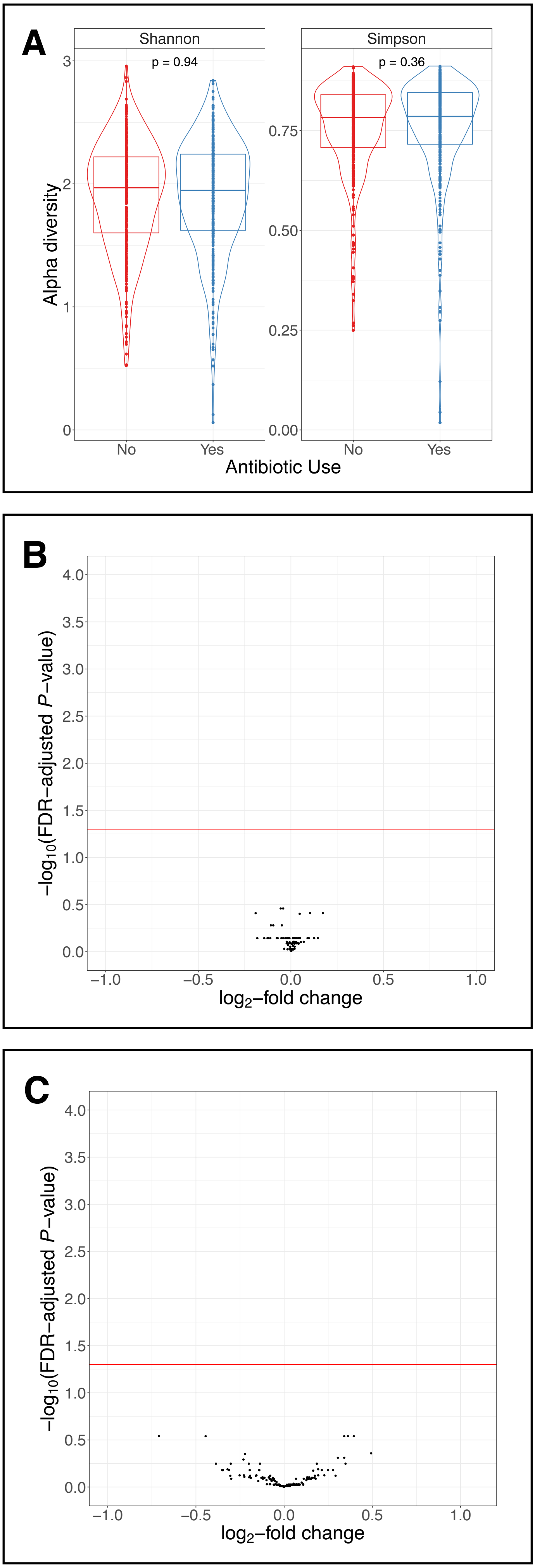

A
Shannon
Simpson
p = 0.94
p = 0.36
Alpha diversity
Antibiotic Use
No
Yes
No
Yes
0
1
2
3
0.00
0.25
0.50
0.75
B
−log10(FDR−adjusted P−value)
log2−fold change
C
−log10(FDR−adjusted P−value)
log2−fold change

Figure S3

A: Volcano plot illustrating the interaction between BRISC trial arm (iron/MNPs and placebo) and antibiotic use in the preceding 7 days in relation to differential abundance of AMR genes (Shotgun metagenomic data). **B**: Volcano plot illustrating the interaction between BRISC trial arm (iron/MNPs and placebo) and antibiotic use in the preceding 7 days in relation to taxonomic differential abundance at the genus level (Shotgun metagenomic data). Each figure shows $\log_2$-fold change on the x-axis and the $-\log_{10}$(FDR-adjusted *P*-value) on the y-axis. The horizontal red line marks the level equivalent to an adjusted *P*-value 0.05. AMR gene abundance was measured in reads per kilobase per million sample reads (RPKM). Source data are provided in the Source Data file.

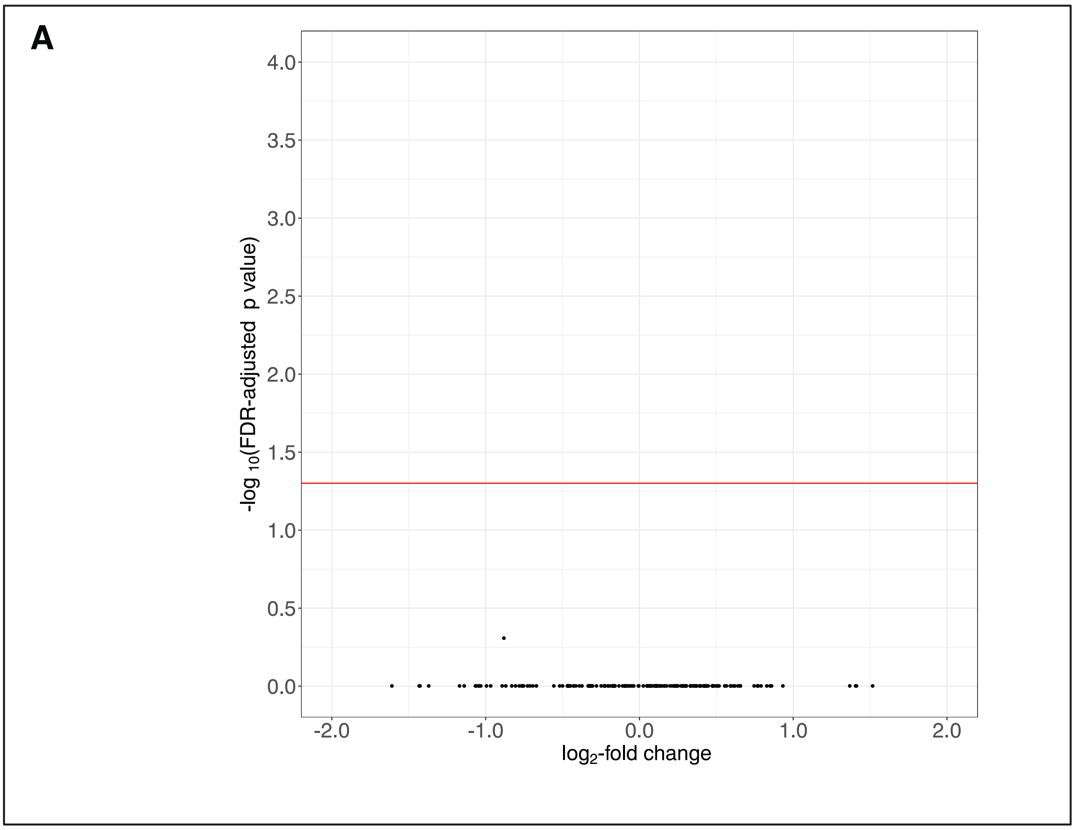
A
4.0
3.5
3.0
2.5
2.0
1.5
1.0
0.5
0.0
-log 10(FDR-adjusted p value)
-2.0
-1.0
0.0
1.0
2.0
log2-fold change

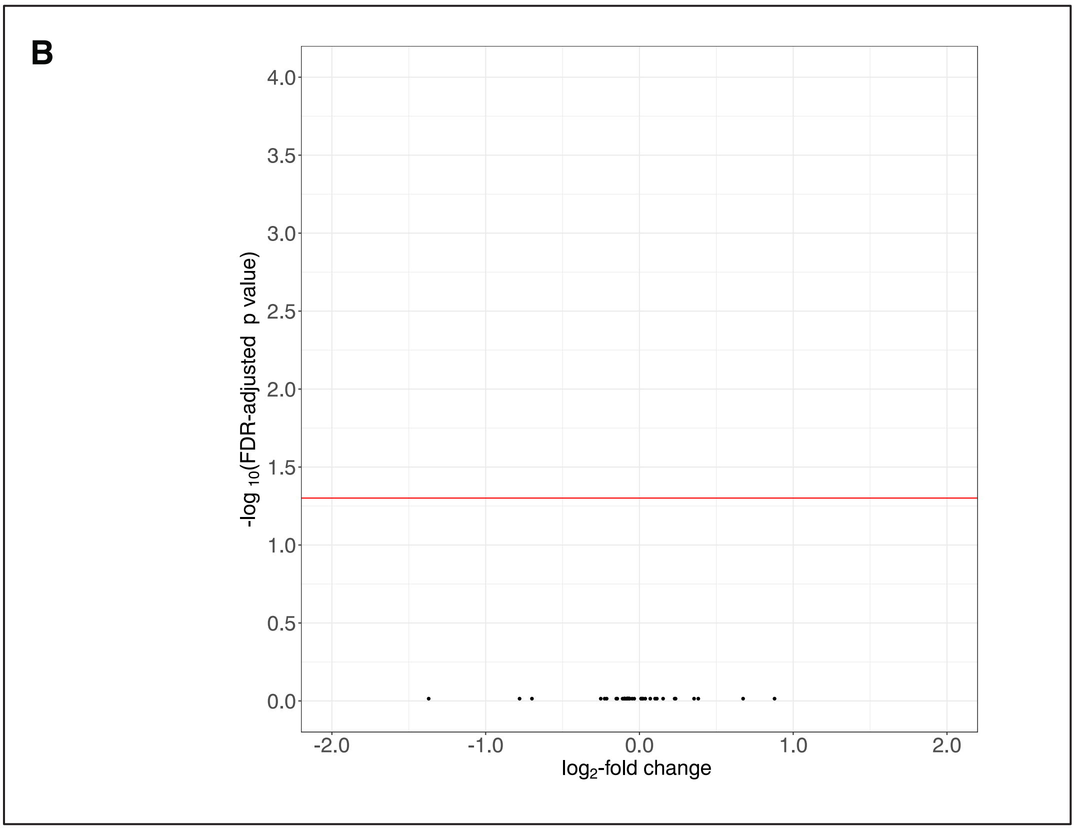
B
4.0
3.5
3.0
2.5
2.0
1.5
1.0
0.5
0.0
-log 10(FDR-adjusted p value)
-2.0
-1.0
0.0
1.0
2.0
log2-fold change

Figure S4

Relative abundance of selected genera by age. **A**: Relative abundance of *Escherichia* by age showing statistically significant reductions in abundance between 8 and 20 as well as between 11 and 20 months of age. **B**: Relative abundance of *Enterococcus* by age showing statistically significant reductions in abundance between all three sampling time points. (Shotgun metagenomic data). n = 316 at 8 months, 316 at 11 months and 312 at 20 months. Source data are provided in the Source Data file.

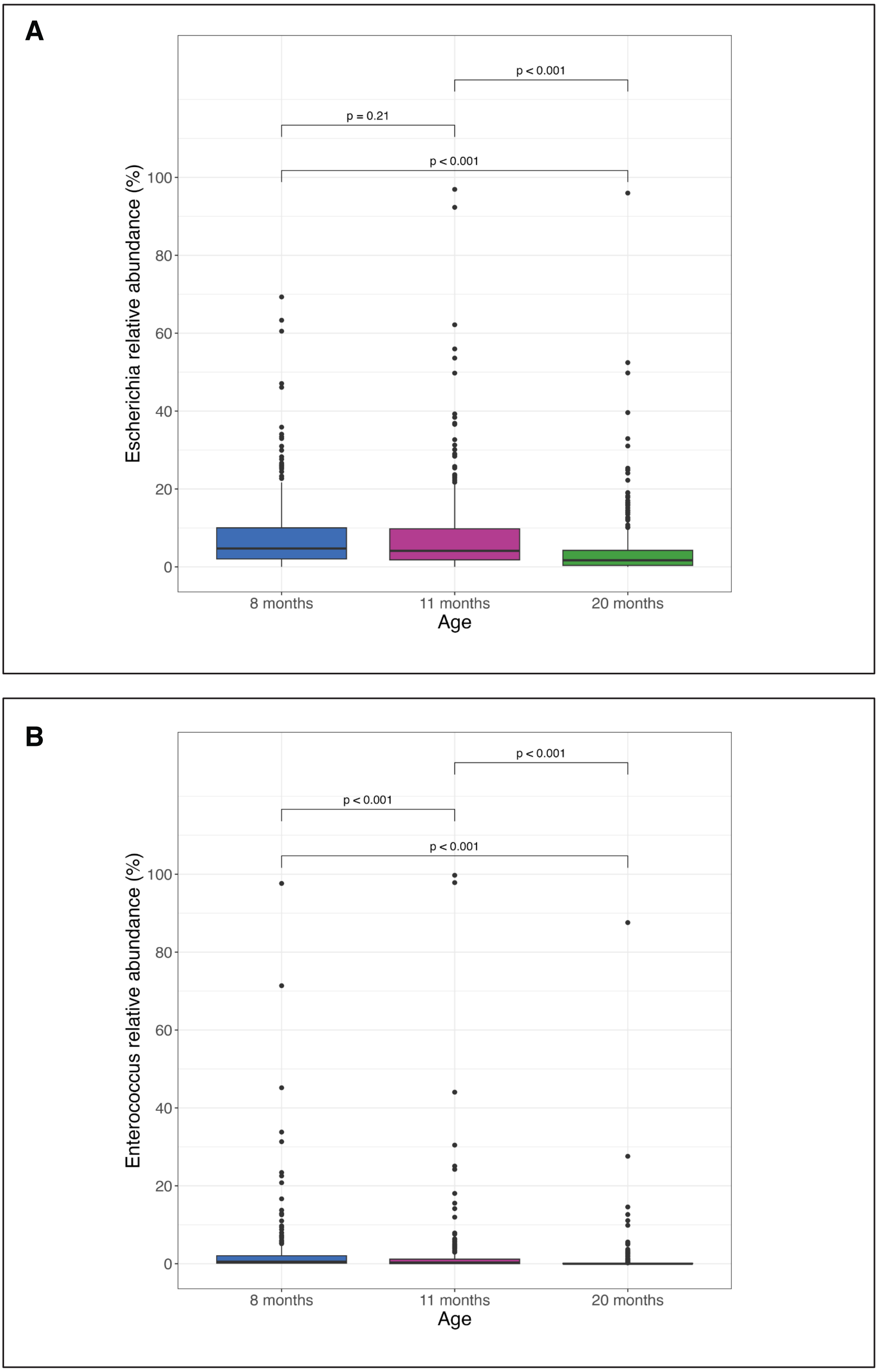
A
p < 0.001
p = 0.21
p < 0.001
Escherichia relative abundance (%)
100
80
60
40
20
0
8 months
11 months
20 months
Age
B
p < 0.001
p < 0.001
p < 0.001
Enterococcus relative abundance (%)
100
80
60
40
20
0
8 months
11 months
20 months
Age

16S rRNA V4 primers and PCR conditions

Primer 515F-OH1 GTGACCTATGAACTCAGGAGTCGGACTACNVGGGTWTCTAAT

Primer 806R-OH2 CTGAGACTTGCACATCGCAGCGTGYCAGCMGCCGCGGTAA

PCR1: 94°C for 3 minutes; 20 cycles at 94°C for 45 seconds each; 55°C for 1 minute; 72°C for 90 seconds. Final step at 72°C for 10 minutes. Amplicons were diluted 1 in 10 before PCR2 (with reaction conditions as above, except an increase to 25 cycles.
