# Supplementary material for: Community use of oral antibiotics transiently reprofiles the intestinal microbiome in young Bangladeshi children
Source: Nat Commun. 2024 Aug 14;15:6980. doi: 10.1038/s41467-024-51326-5 (PMC11324872; doi:10.1038/s41467-024-51326-5)
Supplement: Supplementary file 3 — Reporting Summary [file 41467_2024_51326_MOESM3_ESM.pdf]

nature portfolio

Corresponding author(s): Andrew Baldi
Sant-Rayn Pasricha

Last updated by author(s): 19 Jul 2024

Reporting Summary

Nature Portfolio wishes to improve the reproducibility of the work that we publish. This form provides structure for consistency and transparency in reporting. For further information on Nature Portfolio policies, see our Editorial Policies and the Editorial Policy Checklist.

Statistics

For all statistical analyses, confirm that the following items are present in the figure legend, table legend, main text, or Methods section.

n/a	Confirmed	
☐	☒	The exact sample size (*n*) for each experimental group/condition, given as a discrete number and unit of measurement
☐	☒	A statement on whether measurements were taken from distinct samples or whether the same sample was measured repeatedly
☐	☒	The statistical test(s) used AND whether they are one- or two-sided<br>*Only common tests should be described solely by name; describe more complex techniques in the Methods section.*
☐	☒	A description of all covariates tested
☐	☒	A description of any assumptions or corrections, such as tests of normality and adjustment for multiple comparisons
☐	☒	A full description of the statistical parameters including central tendency (e.g. means) or other basic estimates (e.g. regression coefficient) AND variation (e.g. standard deviation) or associated estimates of uncertainty (e.g. confidence intervals)
☐	☒	For null hypothesis testing, the test statistic (e.g. *F*, *t*, *r*) with confidence intervals, effect sizes, degrees of freedom and *P* value noted<br>*Give P values as exact values whenever suitable.*
☒	☐	For Bayesian analysis, information on the choice of priors and Markov chain Monte Carlo settings
☒	☐	For hierarchical and complex designs, identification of the appropriate level for tests and full reporting of outcomes
☐	☒	Estimates of effect sizes (e.g. Cohen's *d*, Pearson's *r*), indicating how they were calculated

Our web collection on statistics for biologists contains articles on many of the points above.

Software and code

Policy information about availability of computer code

Data collection	Sequencing was performed using the Illumina MiSeq (16S rRNA amplicon sequencing) and Illumina NovaSeq (shotgun metagenomic sequencing) instruments.
Data analysis	16S rRNA amplicon sequencing data was analyzed in RStudio (2023.12.0+369) using packages phyloseq (1.48.0), DADA2 (1.32.0, using SILVA 16S database [NR SSU Ref dataset 99 v138], ggplot2 (3.5.1), microbiomeSeq and ANCOM-BC (2.6.0).<br><br>Shotgun metagenomic sequencing data was analyzed using the bioBakery suite of packages (including KneadData, MetaPhlAn 4.0, ShortBRED and MaAsLin2).

For manuscripts utilizing custom algorithms or software that are central to the research but not yet described in published literature, software must be made available to editors and reviewers. We strongly encourage code deposition in a community repository (e.g. GitHub). See the Nature Portfolio guidelines for submitting code & software for further information.

Data

Policy information about availability of data

All manuscripts must include a data availability statement. This statement should provide the following information, where applicable:

- Accession codes, unique identifiers, or web links for publicly available datasets
- A description of any restrictions on data availability
- For clinical datasets or third party data, please ensure that the statement adheres to our policy

The sequencing data used in this study have been deposited in the NCBI Sequence Read Archive (SRA) database under BioProject accession PRJNA1081952 [https://www.ncbi.nlm.nih.gov/sra].

Code used to process sequencing files (in R for 16S rRNA files and using Python for shotgun metagenomic files), perform statistical analysis and generate figures (in R) is based on the respective packages cited in the manuscript. Custom code was not used. However, code is available from the authors by request.

Research involving human participants, their data, or biological material

Policy information about studies with human participants or human data. See also policy information about sex, gender (identity/presentation), and sexual orientation and race, ethnicity and racism.

Reporting on sex and gender	Analysis of samples from this study was not performed according to sex or gender. Sex was included as a categorical variable in the main BRISC study, but was not used in the microbiome analysis.
Reporting on race, ethnicity, or other socially relevant groupings	Participants were recruited from three Unions (Rupganj, Bhulta and Golakandail) in an area of rural Bangladesh (Rupganj Upazila, ~50km northeast of Dhaka). Socioeconomic data was also collected (parent/guardian-reported), but was not used in the microbiome analysis.
Population characteristics	Participants in this microbiome sub study were recruited at the age of 8 months and provided a stool sample at baseline, 11 months and 20 months. These time points matched the main assessment timepoints of the main study (baseline, immediately post-intervention and post-follow-up). Baseline characteristics are shown in Table 1.
Recruitment	The main BRISC study screened 7.5-8.5-month children in the above unions, excluding those with marked anemia, those with active infection, severe acute malnutrition, developmental delay, and those with a known inherited red cell disorder or previous transfusion. Iron level in drinking water was tested and participants were excluded from the study if iron levels exceeded 1 mg/L.<br><br>This microbiome sub study recruited directly from the main BRISC study - inclusion and exclusion criteria were therefore identical, as was the randomization procedure.<br><br>Travel costs for study visits were reimbursed.
Ethics oversight	This study was approved by the ethics committees at both the International Center for Diarrheal Disease Research, Bangladesh, and Melbourne Health, Australia.

Note that full information on the approval of the study protocol must also be provided in the manuscript.

Field-specific reporting

Please select the one below that is the best fit for your research. If you are not sure, read the appropriate sections before making your selection.

[x] Life sciences [ ] Behavioural & social sciences [ ] Ecological, evolutionary & environmental sciences

For a reference copy of the document with all sections, see nature.com/documents/nr-reporting-summary-flat.pdf

Life sciences study design

All studies must disclose on these points even when the disclosure is negative.

Sample size	As this was a retrospective, cross-sectional analysis of the impact of antibiotic use on the microbiome, sample size was not statistically pre-determined. However, the relatively large sample size for 16S rRNA amplicon analysis, combined with the high sequencing depth from shotgun metagenomics, makes this one of the largest microbiome studies undertaken in the field.
Data exclusions	Samples that yielded <500 reads (16S rRNA amplicon sequencing) and/or that failed the sequencing process were excluded from the analysis. Antibiotic use and reported infections were not exclusion criteria.
Replication	Although samples were not generally sequenced in duplicate, PCR plates and sequencing runs contained a combination of some duplicate samples, as well as a healthy donor sample (aliquots from the same sample) and for shotgun metagenomic sequencing a commercial community standard was included in all plates and sequencing runs (ZymoBIOMICS Microbial Community Standards - Zymo Research).

Randomization	Randomization of participants in the main BRISC study was performed using a computer-generated randomization list with block randomization, stratified according to child sex and union, to link sequential participant identification numbers to trial groups. A hard-copy list was used for randomization in the field.<br><br>Note that the antibiotic and AMR-related analysis was undertaken retrospectively according to antibiotic use, irrespective of trial intervention arm. Definitions of antibiotic use in relation to the sampling timepoint were used to conduct the analysis. These are outlined in the Methods section.
Blinding	The main BRISC study was a three-group, double-blind, double-dummy, individually randomized, placebo-controlled trial. As the microbiome sub study was nested within this trial, these attributes were identical.<br><br>Investigators were blinded to group allocation throughout both data collection and analysis. Samples were also sequenced in random order (i.e. PCR plates and sequencing runs included samples from different timepoints) to minimise any batch effects.

Reporting for specific materials, systems and methods

We require information from authors about some types of materials, experimental systems and methods used in many studies. Here, indicate whether each material, system or method listed is relevant to your study. If you are not sure if a list item applies to your research, read the appropriate section before selecting a response.

Materials & experimental systems

n/a	Involved in the study	
☒	☐	Antibodies
☒	☐	Eukaryotic cell lines
☒	☐	Palaeontology and archaeology
☒	☐	Animals and other organisms
☐	☒	Clinical data
☒	☐	Dual use research of concern
☒	☐	Plants

Methods

n/a	Involved in the study	
☒	☐	ChIP-seq
☒	☐	Flow cytometry
☒	☐	MRI-based neuroimaging

Clinical data

Policy information about clinical studies
All manuscripts should comply with the ICMJEguidelines for publication of clinical research and a completedCONSORT checklist must be included with all submissions.

Clinical trial registration	The microbiome sub study was part of the Benefits and Risks of Iron InterventionS in Children (BRISC) trial (Australian New Zealand Clinical Trials Registry number ACTRN12617000660381).
Study protocol	The protocol for the main BRISC study has been published previously:<br>Hasan MI, Hossain SJ, Braat S, et al. Benefits and risks of Iron interventions in children (BRISC): protocol for a three-arm parallel-group randomised controlled field trial in Bangladesh. BMJ Open 2017; 7(11): e018325. (https://doi.org/10.1136/bmjopen-2017-018325)<br><br>The antibiotic/AMR analysis was a post-hoc analysis and not defined in the main study protocol.
Data collection	Microbiome sub study participants were recruited as the last 1093 children on the main BRISC study, over the period September 2018 to February 2019. The last follow-up stool samples were therefore collected in February 2020.
Outcomes	Primary outcome measures were alpha diversity (Shannon and inverse Simpson indices), beta diversity and differential abundance (taxonomy and AMR gene) with respect to reported antibiotic use at the pre-defined time definitions outlined in the Methods section. Secondary outcomes were not pre-defined. However, additional analyses were performed on the data to examine taxonomic-AMR relationships based on the primary outcome findings.

Plants

Seed stocks	*Report on the source of all seed stocks or other plant material used. If applicable, state the seed stock centre and catalogue number. If plant specimens were collected from the field, describe the collection location, date and sampling procedures.*
Novel plant genotypes	*Describe the methods by which all novel plant genotypes were produced. This includes those generated by transgenic approaches, gene editing, chemical/radiation-based mutagenesis and hybridization. For transgenic lines, describe the transformation method, the number of independent lines analyzed and the generation upon which experiments were performed. For gene-edited lines, describe the editor used, the endogenous sequence targeted for editing, the targeting guide RNA sequence (if applicable) and how the editor was applied.*
Authentication	*Describe any authentication procedures for each seed stock used or novel genotype generated. Describe any experiments used to assess the effect of a mutation and, where applicable, how potential secondary effects (e.g. second site T-DNA insertions, mosiacism, off-target gene editing) were examined.*
